# Further Considerations in Childhood-Onset Hypertrophic Cardiomyopathy Genetic Testing

**DOI:** 10.3389/fcvm.2021.698078

**Published:** 2021-06-21

**Authors:** Michelle M. Monasky, Emanuele Micaglio, Silvia Ignaccolo, Carlo Pappone

**Affiliations:** ^1^Arrhythmia and Electrophysiology Department, IRCCS Policlinico San Donato, Milan, Italy; ^2^Vita-Salute San Raffaele University, Milan, Italy

**Keywords:** hypertrophic cardiomyopathy, childhood-onset, genetic testing, monogenic, oligogenic, modifier genes, channel, desmosomal

Hypertrophic cardiomyopathy (HCM) is a condition in which the interventricular septum and the ventricles become enlarged, which could decrease the ability of the heart to effectively pump the blood, potentially leading to heart failure, arrhythmias, and sudden cardiac death (1, 2). The inheritance has been most often described as following an autosomal dominant pattern (3). However, other inherited causes of left ventricular hypertrophy may include Fabry disease (X-linked) and Friedreich's Ataxia (autosomal recessive) (3). Among recessive HCM, since at least 2017, *ALPK3* mutations have been recognized as important causes (4).

HCM can be caused by variants in sarcomeric genes, non-sarcomeric protein-coding modifier genes, and regulatory non-coding RNA genes (5). Sarcomeric proteins are found in the thin or thick filaments or Z-disc. At least 30 genes can be responsible for HCM (6). Recent studies have used Whole Exome Sequencing to expand the HCM panel, resulting in possible new genes (7–9). Syndromic causes of HCM, such as *VARS2* and *FNIP1*, are emerging genes for pediatric patients, identified by targeted clinical exome sequencing (10, 11). Such studies highlighted some new genotype-phenotype correlations and went beyond the simple task of genetics as “helpful for family planning.” In particular, *VARS2* is additionally associated with pulmonary hypertension and hyperlactacidemia, while *FNIP1* is additionally associated with B cell line immune deficiency and agammaglobulinemia.

We read with great interest a recently published report by Marston et al. (12) investigating the natural history of childhood-onset HCM, the impact of the genetic background, and comparing outcomes to adult-onset disease. HCM may present clinically during all phases of life, yet a higher risk of developing life-threatening ventricular arrhythmias or requiring a transplant or vascular assist device have been associated with earlier disease onset.

Marston et al. (12) performed genetic testing focusing on eight sarcomeric genes. Additionally, the genetic analysis of the genotyped cohort carried out by Marston et al. (12) still refers to HCM as a monogenic disease. Today, the traditional Mendelian fashion of inheritance has been replaced by the oligogenic inheritance model (13, 14), meaning that a mutational load can cause the HCM phenotype. Unfortunately, it is more difficult to provide constructive genetic counseling and help with family planning, understanding that HCM is an oligogenic disease with a complex multifactorial inheritance. Multiple pathogenic genetic variants may be distributed among the family members, which may result in an imperfect co-segregation with the phenotype (13). Modifier genes can play an important role in the expression of the phenotype (15). It is very important to see the family segregation analysis to understand the penetrance.

Moreover, in a nationwide study of Finnish patients (16), 49 variants of unknown significance (VUS) in 31 genes were detected in 20.4% of cases. Marston et al. (12) excluded patients with VUS. It would have been useful to know what VUS had been identified and which criteria had been employed to categorize them as such. VUS should not be interpreted as benign or not noteworthy, but rather as variants about which we do not yet have enough information to understand. Often, there is a wealth of knowledge to be gained from the study of VUS, and, in many cases, variants classified as VUS turn out to be pathogenic, affecting the recommendations made to a person/family (6). However, when it comes to clinical application, it is difficult to determine the combined effect of multiple “VUS,” considering that often only single patients or small pedigrees are examined, making segregation studies not feasible. Also, the ethnicity of the patients need to be considered when assessing the pathogenicity of rare VUS, as ethnicity is a factor in HCM (17). According to us, the HCM genetic panel should include only the established HCM genes for diagnostic purposes, especially in pre-symptomatic situations. We think that the OMIM (18) and Gene Reviews (19) websites can provide useful indications for clinicians. On the other hand, a too restrictive Next Generation Sequencing (NGS) panel might prevent the progress of scientific knowledge in the field of HCM. For this reason, we think that the Comprehensive Metabolic Panel, or an “enlarged” panel, encompassing both arrhythmia and metabolic genes, can be very important for research purposes. If both (diagnostic and research) NGS panels provided a negative result, exome studies would be a choice, with careful considerations about possible incidental results and variants of uncertain significance.

Regarding again the study by Marston et al. (12), it was unclear how “clinical significance” was defined when describing patients with “no clinically significant sarcomeric variants” who were classified as non-sarcomeric HCM. Furthermore, it would have been interesting to know what variants in non-sarcomeric proteins were excluded entirely from the study. Even minor genes identified might have shed more light on childhood-onset HCM, which is not well-characterized given the rarity of the phenotype—something which may correlate with the rarity of a particular genotype.

Finally, given the greater risk of developing life-threatening ventricular arrhythmias with childhood-onset HCM, it is important to consider the possibility of disease overlap in this subpopulation. Genes encoding channel and desmosomal proteins could be investigated. For example, the most commonly found mutated gene in Brugada syndrome is *SCN5A*, encoding for the sodium voltage-gated channel alpha subunit 5, and this gene's protein product is altered in HCM (20). Additionally, a transcriptional misregulation of *SCN5A* has been found in HCM patients even in the absence of *SCN5A* mutations (20). Moreover, heterozygous *SCN5A* variants with a putative negative effect on mRNA splicing have been found in a HCM case series (21). Thus, genes known to be altered in various channelopathies could be a subject of future research.

In conclusion, modifier genes and a possible oligogenic inheritance pattern should be considered when screening patients for HCM. Variants of unknown significance should be carefully evaluated for possible pathogenicity, and genes encoding channel and desmosomal proteins should be considered.

## Author Contributions

MM and EM conceived, wrote, and revised the manuscript. SI provided useful comments. CP provided useful feedback and provided resources and funding. All authors contributed to the article and approved the submitted version.

## Conflict of Interest

The authors declare that the research was conducted in the absence of any commercial or financial relationships that could be construed as a potential conflict of interest.
